# Effects of Dietary Supplement of Probiotic *Enterococcus faecium* on Intestinal Microbiota and Barrier Structure, Immune Function, and Antioxidant Capacity of Soft-Shelled Turtle *Pelodiscus sinensis*

**DOI:** 10.1155/anu/8066906

**Published:** 2025-02-07

**Authors:** Yu Zhang, Yang Lu, Yi Zhang, Cuijuan Niu

**Affiliations:** Ministry of Education Key Laboratory for Biodiversity Science and Ecological Engineering, College of Life Sciences, Beijing Normal University, Beijing 100875, China

**Keywords:** antioxidant defense, *Enterococcus faecium*, immunity, intestinal barrier structure, intestinal microbiota, soft-shelled turtle

## Abstract

*Enterococcus faecium* inhabits animal gastrointestinal tracts and has been demonstrated to benefit livestock and poultry, but its effects on soft-shelled turtles remain unexplored. The present work investigates the effects of probiotic *E. faecium* on intestinal microbiota and barrier structure, immune function, and antioxidant capacity of the soft-shelled turtle. Twenty-four juvenile *Pelodiscus sinensis* were divided into two groups: control (fed a basal diet) and treatment (fed a diet supplemented with *E. faecium*, 3.3 × 10^8^ CFU/g feed), over a period of 6 weeks. We found that *E. faecium* did not promote the growth of turtles at the present feeding level, but the treatment resulted in significant alterations in the intestinal microbial community structure, with increased abundance of *Enterococcus*, *Romboutsia*, and *Clostridium_sensu_stricto_1*, and a reduction in *Aeromonas* (*p*  < 0.05). *E. faecium* notably enhanced villus height/crypt depth, villus width, and villus density in the intestine. The treatment group exhibited a 1.50-fold increase in goblet cells count and a 1.18-fold higher in the muscular layer thickness compared to the control group. *E. faecium* also improved the immune function, with an increase in the ratio of plasma neutrophils and lymphocytes to the total number of leukocytes after feeding probiotics, and upregulation of the levels of toll-like receptor 4 (TLR 4), lysozyme, interleukin 1*β* (IL-1*β*), tumor necrosis factor *α* (TNF-*α*), and immunoglobulin A (IgA) in the intestine, as well as the level of hepatic immunoglobulin M (IgM). Additionally, *E. faecium* supplementation boosted antioxidant capabilities, including a significant increase in catalase (CAT) and glutathione peroxidase (GPx) activity and reduced glutathione (GSH) levels in the intestine and GSH levels in the spleen. Our study demonstrates the beneficial effects of supplemental *E. faecium* on the intestine and overall health of soft-shelled turtles, particularly in enhancing their immune function and antioxidant capacity.

## 1. Introduction

The soft-shelled turtle *Pelodiscus sinensis* is a popular freshwater aquaculture species in China, renowned for its medicinal, culinary, and research value, and has experienced a surge in market demand. However, the rapid expansion of high density, intensive aquaculture has resulted in the degradation of aquatic environments, subsequently contributing to the emergence of bacterial diseases, such as those caused by *Aeromonas*, and led to significant economic losses in the aquaculture of soft-shelled turtles [1]. The intestine is the largest immune and digestive organ in soft-shelled turtles, and the microbial communities residing in the intestine can maintain the integrity of the intestinal physical barrier and activate the host immune system to resist and eliminate invading pathogens when in a state of homeostasis [2]. Hence, it is expected that modulation of the intestinal microbiota can help turtles prevent pathogen invasion [3]. However, there is still limited research on the intestinal microbiota and its role in influencing the physiological characteristics of freshwater turtles. A handful of studies have shown that *P. sinensis* benefits from dietary probiotics, such as *Bacillus subtilis*, by improvement of intestinal health [4, 5]. More research is needed to know the underlying mechanism of exogenous probiotics in improving the soft-shelled turtles' health and to explore more kinds of effective probiotics for heath management of freshwater turtles.

*Enterococcus faecium*, a Gram-positive anaerobic bacterium, dominates the animal intestine. It possesses a diverse range of exceptional biological characteristics, including strong adhesion and colonization abilities, tolerance to gastric acid and bile salts, and a high growth rate [6]. *E. faecium* has been classified as a direct-fed microbial (DFM) by the US Food and Drug Administration (FDA) and Association of American Feed Control Officials (AAFCOs). It finds wide application in piglet and poultry farming and has demonstrated favorable probiotic effects [7], such as improving the structure of intestinal microbiota [8–11], enhancing immune function [12, 13], and promoting growth [14, 15] of the animals. In contrast, there have been fewer studies investigated effects of supplemental *E. faecium* as a probiotic on aquatic animals. Published literature has reported that *E. faecium* can stimulate growth of roach *Rutilus rutilus* and white shrimp *Litopenaeus vannamei* by supplementing the diet with 1 × 10^6^−10^8^ CFU/g feed of it [16, 17], activate the immune system in carp *Cyprinus carpio* at a level of 1 × 10^8^ CFU/g of feed [18], enhance disease resistance in Japanese flounder *Paralichthys olivaceus* by intraperitoneal injection of 1 × 10^9^*E. faecium* cells/ind [19]. Notably, Zhang [20] isolated an *E. faecium* strain DEC-43 from the intestinal tract of *P. sinensis* and supplemented it into diet using different treatment levels from 1 × 10^6^ −10^8^ CFU/g. However, this study just examined the effect of *E. faecium* on nonspecific immune responses in the blood of the soft-shelled turtle [20].

The present study targeted to modify the intestinal microbiota of the soft-shelled turtle by administering exogenous probiotic *E. faecium* and to further investigate its effects on intestinal barrier structure, immune function, and antioxidant capacity of this species.

## 2. Materials and Methods

### 2.1. Diet Preparation

Commercial basal feed used in this experiment was purchased from Zhejiang Xinxin Feed Company (Jiaxing, Zhejiang, China). Main nutritional composition of the basal feed is listed in Table 1, based on the manufacturer's information.

In this study, the probiotic strain was sourced from microencapsulated *E. faecium* RS047 provided by the Academy of National Food and Strategic Reserves Administration (Beijing, China). The concentration of viable bacteria in this commercial microencapsulation preparation was 1 × 10^10^ CFU/g [21]. Based on supplemental recommendation of the supplier, our preliminary experiments and literature reference, considering the feeding rate and feed loss during the acclimation period, the final diet fed to the animals contained probiotics at a concentration of 3.3 × 10^8^ CFU/g, which is a level that already demonstrated the efficacy of *E. faecium* supplementation in various animals [16–20].

Method for preparing *E. faecium* treatment diet was as follows: the basal feed powder and the microencapsulated probiotic particles (brown granules) were mixed in a household dough mixer (HMJ-A35M1, Bear Electric Appliance Co., China) at a speed of 170 rpm for 5 min to ensure uniform distribution of the microencapsulated *E. faecium*. Subsequently, water was added to the feed in a 1:2 (v/w) ratio to produce feed dough. To maintain probiotic viability, the feed containing *E. faecium* was freshly prepared daily before feeding, and the microencapsulated probiotics were stored at 4°C. Additionally, to ensure that the basal feed was free of *E. faecium*, the microbial composition of the feed was regularly tested during the experiment to confirm the absence of the target probiotic in the basal feed. The procedure involved mixing 1 g of basal feed with 9 mL of sterile phosphate buffer solution (PBS), followed by centrifugation to collect the supernatant. A portion of the supernatant was streaked onto LB agar plates using an inoculating loop and incubated at 37°C for 24 h. The cultured bacteria were then isolated, purified, and subjected to 16S rRNA sequencing to determine the presence of *E. faecium* in the feed.

### 2.2. Animal Feeding, Experimental Design, and Sample Collection

In October 2023, *juvenile* soft-shelled turtles (no gender differentiation) were obtained from a turtle hatchery located in Xinyang, Henan Province, China (E114°08.289′; N32°12.862′). The turtles were allocated to six tanks (length × width: 55 cm × 38 cm, water depth: 10 cm), ensuring that individuals within each tank were of similar size. Before the formal experiment, all turtles underwent a 4-week acclimation period. During acclimation, the turtles were fed a commercial basal diet at 3% of their body weight daily in the morning. Any uneaten feed and feces were promptly removed 2 h after feeding, and approximately half of the water in each tank was refreshed daily. Environmental and water quality parameters during the acclimation and experimental periods were maintained as follows: temperature, 28.0 ± 1.0°C; dissolved oxygen, 6.317 ± 0.079 mg/L; pH, 7.295 ± 0.067; total ammonia nitrogen, <0.2 mg/L; and light conditions, natural light.

After the 4-week acclimation, 24 healthy turtles (average body weight 105.05 ± 4.57 g) were randomly divided into two groups: control and treatment group. The basal diet for the control group did not contain *E. faecium*, while the treatment group received feed supplemented with microencapsulated *E. faecium*. Feeding methods and environmental conditions during the experimental period were the same as those during acclimation. To ensure equal access to food, solid feed was evenly distributed across multiple locations within each tank.

At the end of the experiment, turtles were euthanized by rapid decapitation and dissected on ice. Blood samples were collected from the neck–chest junction using heparinized tubes. A portion of the blood was left in an ice bath for 2 h and then centrifuged at 4°C and 3000 r/min for 10 min to separate the serum. The remaining blood was used on the same day to prepare whole blood smears. Additionally, the liver, intestine, and spleen were rapidly excised and immediately frozen in liquid nitrogen. Serum and tissue samples were stored at −80°C for subsequent analyses. This experiment was conducted in strict accordance with the standards of the Ethical and Animal Welfare Committee (EAWC) of Beijing Normal University, with approval number CLS-EAW-2020-016.

### 2.3. Growth Performance

Both at the beginning and end of the experiment, after a 24-h fasting period, body weight of each experimental turtle was measured, respectively, then the following parameters were assessed according to the method described by Shija et al. [22]:

  $$\begin{array}{c} \text{Survival rate}\left(\text{SR},\%\right)=100\times \text{final number/initial number}, \end{array}$$

  $$\begin{array}{c} \text{Weight gain rate }\left(\text{WGR},\%\right)=100\times \left(\text{final weight (g) − initial weight (g)}\right)/\text{initial weight (g)}, \end{array}$$

  $$\begin{array}{c} \text{Specific growth rate }\left(\text{SGR},\;\%/d\right)=100\times \left[\ln\;\left(\text{final weight }\left(g\right)\right)-\ln\;\left(\text{initial weight }\left(g\right)\right)\right]/\text{time of the experiment covered}. \end{array}$$

### 2.4. Intestinal Microbiota Analysis

The microbiota was determined in the small intestine of 10 individuals randomly taken from each group. The intestine was aseptically collected, opened, and all the intestinal contents were gently scraped with a sterile scalpel, and microbial DNA was extracted using the DNA Kit (TianGen, China). Purity and concentration were assessed via 1% agarose gel electrophoresis. The V3-V4 hypervariable regions of the bacterial 16S rDNA gene were amplified using specific primer pairs: forward 5′−3′ (CCTAYGGGRBGCASCAG) and reverse 5′−3′ (GGACTACNNGGGGTATCTAAT). Each PCR reaction mixture contained 15 μL PCR Master Mix (New England Biolabs), 2 µM primers, and 10 ng template DNA. The program included an initial denaturation at 98°C for 1 min, followed by 30 cycles of denaturation at 98°C for 10 s, annealing at 50°C for 30 s, and elongation at 72°C for 30 s, with a final extension at 72°C for 5 min. PCR products passing electrophoresis testing were purified using the Universal DNA Purification Kit (TianGen, China). Libraries were prepared using the DNA Library Prep Kit (New England Biolabs, USA), quantified with Qubit and real-time PCR, and pooled for sequencing on the NovaSeq 6000 system. Sequenced data underwent assembly, filtering, and alignment to remove chimeras.

QIIziseas utilized for species annotation [23] to identify the top 10 species based on relative abundance at the genus level. *α*-diversity indexes (Shannon and Simpson) were performed by *t*-test. *β*-diversity was assessed via unifrac distance and unweighted unifrac distance, followed by principal co-ordinates analysis (PCoA). Linear discriminant analysis effect size (LEfSe) software was used to identify the specific microbiota linked to *E. faecium* treatment with a default LDA score threshold of 4. The MetagenomeSeq method was used to conduct hypothesis tests on intergroup species abundance data, and species with significant intergroup differences were then selected based on *p*-values (*p* < 0.05).

### 2.5. Intestinal Barrier Structure

Intestine segments were fixed with 4% paraformaldehyde and embedded in paraffin, which was stained with hematoxylin and eosin (HE) for general morphology and Alician Blue-Periodic Acid Schiff (AB-PAS) for identification of goblet cells. Each sample underwent microscopic examination, where villi were analyzed for villus height (from the tip of the villus to the villus–crypt junction), crypt depth (from the bottom of the villus to the lamina propria), the villus height-to-crypt depth ratio and villus width (between the two endpoints at the base of the villus) (Figure S1a). Additionally, random fields of view from each sample were selected for assessing villus density (the number of complete intestinal villi in each field) and measuring the thickness of the submucosal and muscular layers at their widest point using ImageJ software. The number of goblet cells was counted on 100 columnar cells of villus mucosa for each complete villus in the view [24] (Figure S1b).

### 2.6. Immunological Assays

To assess immune function, we collected serum, liver, and intestine from each individual to measure immune indicators, including lysozyme (LZM), interleukin 1*β* (IL-1*β*), tumor necrosis factor *α* (TNF-*α*), toll-like receptor 4 (TLR 4), complement 3 (C3), complement 4 (C4), immunoglobulin A (IgA), immunoglobulin M (IgM), and immunoglobulin G (IgG). Notably, owing to the spleen's small size and no enough samples for the assay, we did not assess immunological indices of this tissue. Serum LZM was measured using assay kits (Shanghai Tongwei, China), which employed photometric changes caused by the degradation of a turbid bacterial solution. IL-1*β*, TNF-*α*, TLR 4, C3, C4, IgA, IgM, and IgG were detected with commercial ELISA (FANKEL Industrial Co., Ltd., Shanghai, China) as per the manufacturer's instructions. Standards and diluted samples (50 µL) with varying concentrations were sequentially added to the ELISA plate. After incubation at 37°C for 30 min, the plate was washed five times with washing water. Subsequently, 50 µL of horseradish peroxidase (HRP) coupling reagent was added to each well, incubated at 37°C for 30 min, and the plate was washed five times again. Following this, 50 µL of color development reagents A and B were added. After incubating for 10 min, the reaction was stopped by adding 50 µL of termination solution. The optical density value was measured at 450 nm by a microplate reader, and the concentration of each immunological factor was calculated based on the standard curve. Blood smears were made from the whole blood and stained with Wright–Giemsa to determine the relative percentages of heterophils, lymphocytes, monocytes, eosinophils, and basophils in 100 leukocytes.

### 2.7. Antioxidant Capacity and Protein Assessment

Liver, intestine, and spleen were homogenized on the ice in PBS at a ratio 1:9. Then, the homogenates were centrifuged, and the supernatants were collected. Enzyme activities of superoxide dismutase (SOD), catalase (CAT), glutathione peroxidase (GPx), reduced glutathione (GSH), as well as protein, malondialdehyde (MDA), and total antioxidative capacity (T-AOC) levels, were conducted by the assay kits according to the instructions (Nanjing Jiancheng Bioengineering Institute, China). The absorbance values were measured under a TECAN Spark full-band microplate luminescence detector.

SOD activity was obtained by measuring the cytochrome c reduction inhibition reaction of xanthine–xanthine oxidase by the absorption value at 550 nm. CAT activity was assessed by evaluating the extent of hydrogen peroxide (H_2_O_2_) decomposition. GPx activity was expressed by the reaction rate of catalyzing GSH. GSH reacts with DTNB [5,5′-dithio-bis- (2-nitrobenzoic acid)] to generate TNB (2-nitro-5-thiobenzoic acid), which can be measured at 412 nm. The level of MDA was determined based on the reaction of MDA and 2-thiobarbituric acid. Antioxidants in the tissues facilitated the reduction of Fe^3+^ to Fe^2+^. T-AOC was evaluated via the absorbance of a stable complex formed by phenanthrolines and Fe^2+^. Total protein content was quantified in accordance with the bicinchoninic acid (BCA) method.

### 2.8. Statistical Analysis

Statistical analysis was performed using SPSS 23.0. All data are presented as mean ± SE, with *p* < 0.05 accepted as a significant difference. Normality and homogeneity of variances were assessed through the Shapiro–Wilk test and Levene's test, respectively. Independent samples *t*-test was used for comparative analysis between the groups when both assumptions were met. The Mann–Whitney *U* hoc test was employed if the data showed unsatisfactory normality.

## 3. Results

### 3.1. Growth Performance

As shown in Table 2, no animals died during the entire experimental period in either group. Therefore, the SR was 100% in both groups. Additionally, there were no significant differences between the control group and the treatment group in terms of final weight (Wt, *p* = 0.534), WGR (*p* = 0.887), and SGR (*p* = 0.887).

### 3.2. Intestinal Microbiota

**Figure 1:**
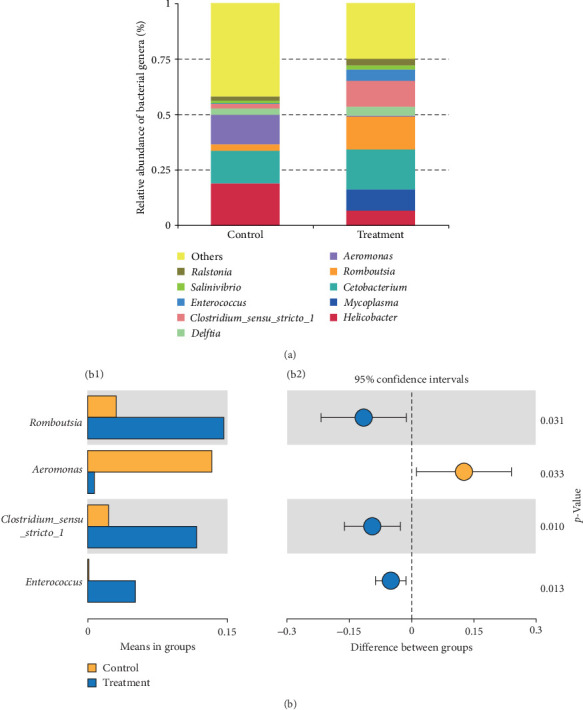
Comparison of intestinal microbiota of the soft-shelled turtles treated with different diets at genus level. (a) Relative abundance of the top 10 most abundant bacterial genera in the intestinal microbiota of the control and treatment groups. Genera with lower abundances were grouped under “others.” (b) Species with significant differences between groups by *t*-test at genus level. Panel (b1) shows species abundance differences between groups, with bars representing group means. Panel (b2) depicts confidence levels for intergroup variations, with circles indicating mean differences and lines showing 95% confidence intervals.

**Figure 2:**
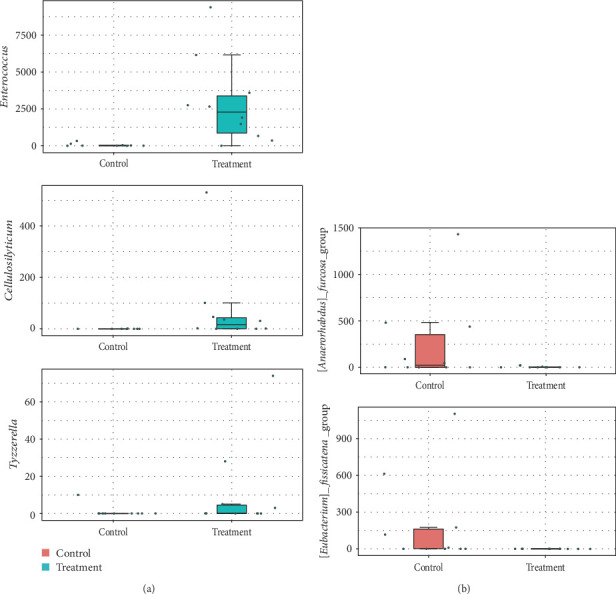
Species with significant differences between groups by MetagenomeSeq at genus level. *⁣*^*∗*^*p*  < 0.05. (a) Species with significantly increased abundance in the treatment group; (b) Species with significantly decreased abundance in the treatment group.

A total of 1,976,762 clean reads were obtained with high Q20 and Q30 (>96%) after quality trimming, indicating good sequencing quality of sequencing data (Table S1). Rarefaction analysis indicated that sufficient sequencing depth was achieved for all samples (Figure S2). *α*-diversity indexes (Shannon and Simpson) between groups showed no significant differences (*p*  > 0.05, Figure S3a). PCoA analysis revealed no significant differences in *β*-diversity based on weighted and unweighted UniFrac distances (*p*  > 0.05, Figure S3b). Firmicutes were the predominant species that increased in the treatment group, including Peptostreptococcaceae, Clostridiales, and Enterococcaceae at the family level based on LEfSe analysis. Compared to the control group, the treatment group exhibited a significant decrease in Rikenellaceae and Enterobacterales (*p*  < 0.05, Figure S4). Among the top 10 genera by relative abundance, *Aeromonas* showed a significant decrease (*p*  < 0.05, Figure 1a,b), while *Enterococcus*, *Romboutsia*, and *Clostridium_sensu_stricto*_1 significantly increased after probiotic administration (*p*  < 0.05, Figure 1a,b). Following *z*-standardization treatment, only *Enterococcus* exhibited a significant increase in the probiotic-supplemented group at genus levels (*p*  < 0.05, Figure S5). Using MetagenomeSeq to identify species with significant intergroup differences at the genus level, notable increases were observed in *Enterococcus*, *Cellulosilyticum*, and *Tyzzerella* (*p*  < 0.05, Figure 2a), while [*Anaerorhabdus*] *furcosa*_group and [*Eubacterium*] *fissicatena*_group decreased in the intestine (*p*  < 0.05, Figure 2b).

### 3.3. Intestinal Barrier Structure

**Figure 3:**
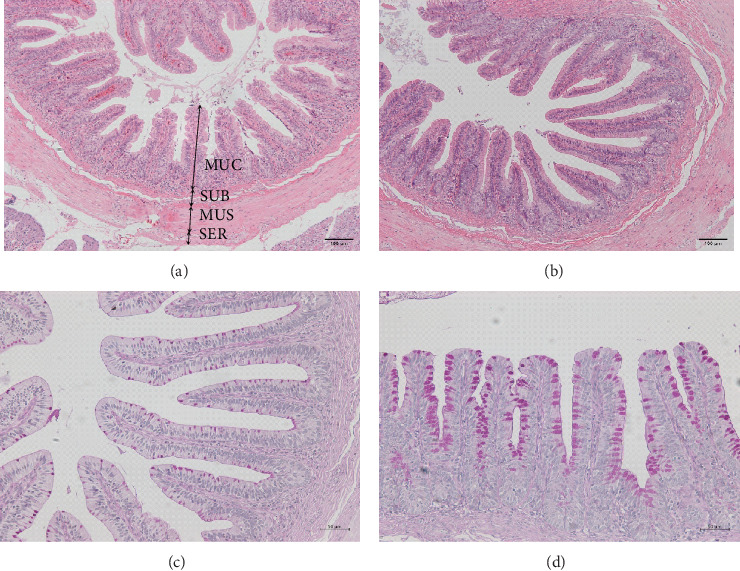
Effects of *E. faecium* on intestinal morphology of the soft-shelled turtles. (a, b) Representative micrographs of the intestinal morphology carried out on sections stained with HE from the small intestine (100x magnification). MUC, mucosal layer; MUS, muscular layer; SER, serosal layer; SUB, submucosal layer. (c, d) Effects of *E. faecium* on goblet cells in intestine of the soft-shelled turtles. Representative micrographs of goblet cell staining carried out on sections strained with AB-PAS from the small intestine (50x magnification). a/c, control; b/d, treatment.

**Figure 4:**
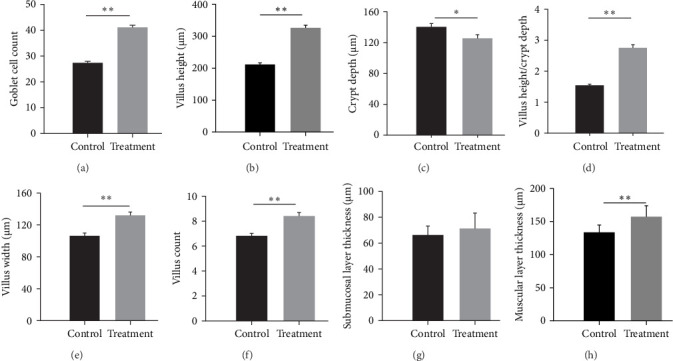
Effects of *E. faecium* on intestinal barrier of the soft-shelled turtles. (a) Goblet cell count; (b) Villus height; (c) Crypt depth; (d) Villus height/Crypt depth; (e) Villus width; (f) Villus count; (g) Submucosal layer thickness; (h) Muscular layer thickness. Data are presented in mean ± SE (*n* = 6). Asterisks indicate significant differences: *⁣*^*∗*^*p* < 0.05, *⁣*^*∗∗*^*p* < 0.01.

The intestinal wall comprises four layers: the mucosal, submucosal, muscular, and serosal layers, arranged from the innermost to the outermost (Figure 3a). The control group displayed loosely arranged intestinal villi, accompanied by a partial detachment of epithelial cells. In contrast, the treatment group exhibited tightly arrayed villi with a neat striated border (Figure 3a,b), and a 1.50-fold increased amount of goblet cells was detected in this group compared with the control (*p* < 0.001, Figures 3c,d and 4a). Additionally, the probiotic-fed group showed a substantial increase in villus height (*p* < 0.001, Figure 4b) and decrease in crypt depth (*p* = 0.018, Figure 4c), resulting in a remarkable 1.78-fold increase in the villus height/crypt depth ratio compared to the control group (*p* < 0.001, Figure 4d). Villus width and villus count also exhibited a highly significant increase (*p* < 0.001, Figure 4e,f). No significant difference was observed in submucosal layer thickness (*p* = 0.459, Figure 4g), but the muscular layer thickness in *P. sinensis* fed with *E. faecium* increased by 1.18-fold (*p* < 0.001, Figure 4h).

### 3.4. Immune Function

**Figure 5:**
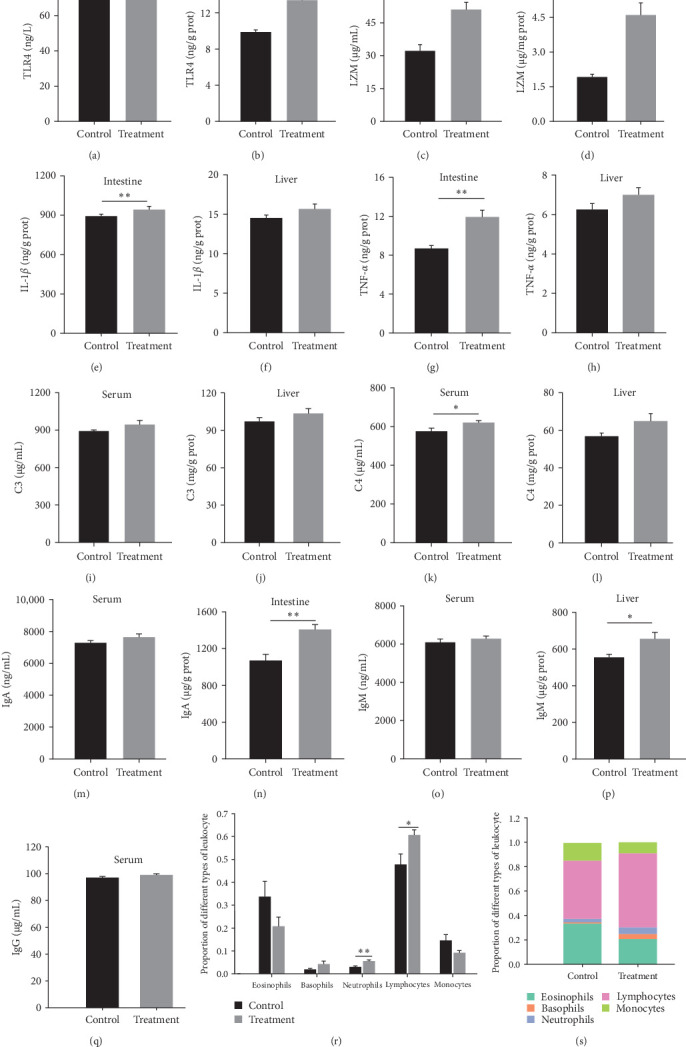
Effects of *E. faecium* on immune function in serum, intestine, and liver of the soft-shelled turtles. (a, b) TLR4 in the serum and intestine; (c, d) lysozyme in the serum and intestine; (e, f) IL-1*β* in the intestine and liver; (g, h) TNF-*α* in the intestine and liver; (i, j) C3 in the serum and liver; (k, l) C4 in the serum and liver; (m, n) IgA in the serum and intestine; (o, p) IgM in the serum and liver; (q) IgG in the serum. Data are presented in mean ± SE (*n* = 8). (*r*, s) Effects of *E. faecium* on different types of leukocyte in blood (*n* = 6). Asterisks indicate significant differences: *⁣*^*∗*^*p* < 0.05, *⁣*^*∗∗*^*p* < 0.01.

TLR4 levels in the intestine were 1.36-fold higher than those in the controls, with no significant changes in serum (*P*_intestinal_ < 0.001; *P*_serum_ = 0.959, Figure 5a,b). The content of LZM in the intestine and serum of the probiotic-fed group was 2.41 and 1.59 fold higher than the control group, respectively (*P*_intestinal_ < 0.001; *P*_serum_ = 0.001, Figure 5c,d). Remarkably elevated levels of intestinal inflammatory cytokines IL-1*β* and TNF-*α* were observed in the treated group, but no significant changes were noted in the liver between the groups (IL-1*β*: *P*_intestinal_ = 0.002, *P*_liver_ = 0.182; TNF-*α*: *P*_intestinal_ = 0.001, *P*_liver_ = 0.147, Figure 5e–h). Apart from the significantly higher serum C4 level in the *P. sinensis* fed with *E. faecium* group compared to the control group (*p* = 0.028), no significant differences were observed in liver complement and serum C3 levels during this experiment (*p*  > 0.05, Figure 5i–l). The IgA levels in the intestine and IgM levels in the liver increased by 1.31-fold and 1.19-fold, respectively, in the treatment group compared to the control, with no significant changes in serum immunoglobulin levels (IgA: *P*_intestinal_ = 0.002, *P*_serum_ = 0.209; IgM: *P*_liver_ = 0.020, *P*_serum_ = 0.472, IgG: *P*_serum_ = 0.265, Figure 5m–q). Comparatively, the proportion of blood neutrophils and lymphocytes over total leukocytes highly increased in the treatment group (*P*_neutrophils_ = 0.010, *P*_lymphocytes_ = 0.039, Figure 5r,s).

### 3.5. Antioxidant Capacity

**Figure 6:**
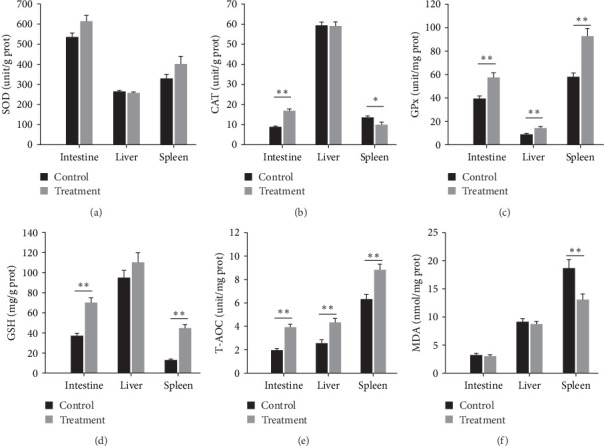
Effects of *E. faecium* on antioxidant capacity in intestine, liver, and spleen of the soft-shelled turtles. (a) SOD; (b) CAT; (c) GPx; (d) GSH; (e) T-AOC; (f) MDA. Data are presented in mean ± SE (*n* = 12). Asterisks indicate significant differences: *⁣*^*∗*^*p* < 0.05, *⁣*^*∗∗*^*p* < 0.01.

In contrast to the control group, no significant differences were identified in SOD activities (SOD: *p* > 0.05, Figure 6a). CAT activity in the treatment group showed a significant increase in the intestine and decrease in the spleen (CAT: *P*_intestinal_ < 0.001, *P*_liver_ = 0.922, *P*_spleen_ = 0.035, Figure 6b). The treatment group also displayed a remarkable increase in GPx activity in the intestine, liver, and spleen (GPx: *P*_intestinal_ = 0.001, *P*_liver_ = 0.005, *P*_spleen_ < 0.001, Figure 6c). Furthermore, there was a noticeable rise in GSH level both in the intestine and spleen (GSH: *P*_intestinal_ < 0.001, *P*_liver_ = 0.226, *P*_spleen_ < 0.001, Figure 6d). The levels of T-AOC in all the tissues were significantly higher than those in the control group (T-AOC: *P*_intestinal_ < 0.001, *P*_liver_ = 0.002, *P*_spleen_ = 0.001, Figure 6e). Moreover, no significant difference in MDA levels in the intestine and liver was observed between groups 11, yet the splenic MDA level became markedly lower than the control (MDA: *P*_intestinal_ = 0.584, *P*_liver_ = 0.589, *P*_spleen_ = 0.008, Figure 6f).

## 4. Discussion

*E. faecium* is an important probiotic widely used in poultry and livestock farming; however, studies on its effects on improving health of the soft-shelled turtles (*P. sinensis*) remained scarce. Our present study demonstrated that supplementation with 3.3 × 10^8^ CFU/g feed of *E. faecium* in the diet improved intestinal microbiota homeostasis and intestinal morphology in *juvenile* turtles while enhancing immune function and antioxidant capacity. However, the impact on growth performance parameters was minimal.

It has been reported that supplementing *E. faecium* can enhance the growth performance of aquatic animals [17, 25]. However, our experiment showed that *E. faecium* supplementation did not significantly affect growth performance of the soft-shelled turtle. This discrepancy may be attributed to the experimental period occurring in winter, which is a season for turtles to hibernate in their natural habitat [26, 27]. Although our rearing water temperature was in a suitable range for growth of the turtles [28], the relatively slow growth of turtles both in the control and treatment groups hints that there may exit an endogenous circannual rhythm suppressed growth or metabolism of the turtles, as the parents of these turtles typically hibernate in the field pond during winter. Further studies are needed to investigate the effects of *E. faecium* on the growth performance of the turtles under varying ecological conditions and across different seasons.

Supplementing probiotics helps maintain a healthy intestinal microbiota, which promotes the growth of animals. Probiotics achieve this by colonizing the intestine or influencing other microbes within the intestinal environment to suppress harmful bacteria, thereby restoring microbiota homeostasis [29]. Our study found that supplementing *E. faecium* had no significant impact on the *α*-diversity of the intestinal microbiota in soft-shelled turtles, as indicated by the Shannon and Simpson indexes. Consistent with our findings, dietary supplementation with *E. faecium* did not affect the Shannon and Simpson diversity indices of piglets' intestinal microbiota [9]. This result may be attributed to the limited effect of probiotics on species diversity in the intestinal microbiota of turtles. However, the richness of some intestinal resident microbial communities changed in the treatment group. Specifically, the level of *Enterococcus* was significantly higher in the treatment group compared to the control, indicating successful colonization of *E. faecium* in the intestine. Additionally, probiotic treatment increased the abundance of several bacterial taxa beneficial for intestinal digestion and metabolism at the phylum or genus level. These included Firmicutes, which maintain microbiota homeostasis [30]; *Romboutsia* and *Clostridium_sensu_stricto_1*, which facilitate energy utilization by intestinal cells [31]; *Cellulosilyticum*, which enhances digestive enzyme and LZM activity [32]; and *Tyzzerella*, which produces anti-inflammatory short-chain fatty acids (SCFAs) [33]. These findings confirm the ability of *E. faecium* to enhance the proportion of beneficial bacteria in the intestine of turtles. Meanwhile, the abundance of several harmful bacterial taxa in the intestine was suppressed in the treatment group. LEfSe analysis revealed significant reductions in the abundance of Rikenellaceae and Enterobacterales, both of which are associated with intestinal inflammation and the expression of proinflammatory factors [34–36]. The abundance of *Eubacterium fissicatena*, known for its role in disrupting the intestinal barrier and inducing colitis [37], also decreased. Additionally, *Aeromonas*, which poses significant harm to soft-shelled turtles [38], showed a notable reduction in abundance in the treatment group. This finding aligns with previous studies showing that supplementing Nile tilapia *Oreochromis Niloticus* feed with *E. faecium* helps combat *Aeromonas hydrophila* infections, further supporting the inhibitory effect of *E. faecium* on *Aeromonas* species [39]. Overall, our study provides preliminary evidence that *E. faecium* modulates the intestinal microbiota of soft-shelled turtles, functioning as antimicrobial agents that increases the proportion of beneficial bacteria while reducing the abundance of harmful ones.

Gut health depends not only on microbiota homeostasis but also on proper intestinal morphology, which is a key factor in ensuring proper digestive function and healthy growth. Within the mucosal layer, villi and crypts are critical morphometric indicators for assessing intestinal development and its digestive and absorptive functions. Increases in villus height and width indicate more intestinal epithelial cells and greater contact with nutrients [40], while shallow crypts suggest faster epithelial cell maturation [41]. Consequently, a higher villus height to crypt depth ratio (V/C) is associated with improved nutrient absorption [42]. Our results confirmed that *E. faecium* administration increased villus height and width, reduced crypt depth, and elevated the V/C ratio. It is also notable that probiotic intake affects villus density [43]. In our study, the increase in villus density further supported the benefits of probiotic supplementation. A robust submucosal layer facilitates nutrient absorption, while a strong muscular layer ensures proper intestinal peristalsis. Xu et al. [44] demonstrated that using biofloc technology to incorporate probiotics substantially increased the thickness of the muscular and submucosal layers in goldfish *Carassius auratus*. Additionally, thicker mucosal and muscular layers enhance resistance against pathogens and inflammation, decreasing the risk of intestinal diseases [45]. Our findings also indicated that feeding *E. faecium* increased thickness of the intestinal muscular layer of the soft-shelled turtle. In summary, exogenous *E. faecium* addition positively influenced intestinal morphology of the soft-shelled turtle.

The resistance of soft-shelled turtle to pathogenic microorganisms is highly dependent on nonspecific immunity, and probiotics have a wide range of regulatory effects on the body's nonspecific immunity [3]. Previous studies have demonstrated that *E. faecium* significantly increases the number of nonspecific immune cells [46]. Similarly, our study confirmed these findings, showing that the number of neutrophils in the blood of the treatment group was significantly higher than that in the control group. This result indicates that *E. faecium* can promote the proliferation of nonspecific immune effector cells in turtles. Various nonspecific immune effector molecules, such as LZM, also play critical roles in defense. Specifically, Paneth cells, located at the base of intestinal crypts, selectively secrete LZM to target pathogens [47], while the peptidoglycan hydrolase secreted by *E. faecium* can stimulate Paneth cells in the intestine to produce LZM, thereby enhancing intestinal immunity [11]. Our studies also showed a marked increase in LZM levels in both the intestine and serum of turtles treated with *E. faecium*. Complement proteins, with strong antimicrobial and immune-activating properties, primarily operate in the bloodstream [48]. Our research confirmed that *E. faecium* raised C4 levels in serum of the soft-shelled turtle. Cytokines, key molecules that identify pathogens and activate the immune system, such as IL-1*β*, TNF-*α*, and IFN-*γ*, are also influenced by probiotics [49]. Our findings further showed that *E. faecium* raised IL-1*β* and TNF-*α* levels in the intestinal tract, enhancing the nonspecific immune barrier, which confirmed the research of Choi et al. [50] on *E. faecium*. Kim et al. [19] observed that feeding *E. faecium* W24 to *Channa argus* led to an upregulation of inflammatory cytokines, including TNF-*α* and IL-1*β*, in multiple tissues. The underlying mechanism may involve the gradual modulation of the intestinal microbiota by *E. faecium* following intestinal colonization, which can reduce the abundance of certain harmful bacterial populations. During this process, mild activation of specific inflammatory signaling pathways, such as the NF-*κ*B and TLR4 pathways, may occur, resulting in elevated levels of pro-inflammatory cytokines [19]. TLR4 is a key TLR family molecule recognizing viruses, fungi, and mycoplasmas that activates intestinal epithelial cells against invading pathogens [51]. *E. faecium* HDRsEf1 enhanced the expression of various TLR molecules in the murine intestine to mitigate barrier damage caused by pathogenic *Escherichia coli* [52]. Our study also confirmed that *E. faecium* enhanced TLR4 level in the intestinal tract of soft-shelled turtles.

Specific immunity is also crucial for the soft-shelled turtle to combat pathogenic microbial invasion, relying primarily on a variety of lymphocytes and immunoglobulins. In general, *E. faecium* influences specific immunity by increasing the number of mucosal epithelial lymphocytes, thereby enhancing resistance to pathogens [53]. Our research further revealed that *E. faecium* elevated lymphocyte counts in the turtle's bloodstream. Moreover, *E. faecium* functions as a specific immunomodulatory agent, stimulating cellular immunity and increasing the levels of various immunoglobulins. The intestinal tract secretes secretory IgA as the first line of defense against pathogens [54], and administration of *E. faecium* SF68 to mice infected with *Giardia intestinalis* significantly raised the proportion of CD^4+^ T lymphocytes in their kidneys, along with increases in fecal IgA and serum IgG levels. Our findings also showed that *E. faecium* enhanced IgA concentration in the turtle's intestine. Additionally, an upregulation of IgM secretion was observed in the liver following treatment. Studies have reported that feeding chickens with fermented products of *E. faecium* raised serum IgM levels, corroborating that *E. faecium* can activate specific immunity [55]. In brief, our results indicate that dietary supplementation with *E. faecium* can enhance both nonspecific and specific immune functions in soft-shelled turtles.

The proper functioning of antioxidant capacity is also critical for maintaining the health of soft-shelled turtles. Various antioxidant enzymes and molecules help reduce the reactive oxygen species (ROS) generated during aerobic metabolism and mitigate the harmful effects they cause. Studies have demonstrated that *E. faecium* possesses potent free radical scavenging capabilities, reducing power and the ability to inhibit ascorbic acid oxidation in vitro [6]. Yu et al. [14] observed that continuous feeding of broiler chickens with diets containing 100 and 200 mg/kg of *E. faecium* for 42 days significantly increased levels of SOD, CAT, and GPx, as well as T-AOC in their pectoral muscles. Our studies also revealed that exogenous *E. faecium* addition significantly enhanced activities of CAT, GPx, and GSH in liver, spleen, and small intestine of the soft-shelled turtle, raising T-AOC levels across all tissues. Furthermore, *E. faecium* reduced the level of the oxidative stress marker, MDA, in the spleen, while MDA levels in other tissues remained unchanged. This specific enhancement of the spleen's antioxidative response is likely due to the probiotic's effect on systemic metabolism, which more effectively eliminates free radicals caused by oxidative stress and minimizes MDA formation. Notably, the spleen's specialized immune cells, such as macrophages and lymphocytes, are particularly abundant and may be more responsive to the metabolic products of probiotics [56]. Similarly, Zhang et al. [57] found that adding butyric acid–producing *Clostridium* to the feed reduced levels of lipid peroxides in the spleen of *Nile tilapia*. In conclusion, *E. faecium* strengthened the antioxidative defense capabilities of the soft-shelled turtle, though the precise mechanisms require further investigation.

## 5. Conclusions

Our results demonstrated that supplement of *E. faecium* RS047 at a concentration of 3.3 × 10^8^ CFU/g feed of soft-shelled turtles optimizes the composition of the intestinal microbiota, particularly by reducing the abundance of harmful bacteria and improving the structure of the intestinal barrier. Compared to previous studies, our study further confirms that *E. faecium* enhances both the nonspecific and specific immunity of soft-shelled turtles and boosts its antioxidant defense capacity. These findings highlight the great potential of *E. faecium* as a probiotic candidate in turtle culture. Further research is needed to determine the optimal dosage and timing for utilization of exogenous *E. faecium* in aquaculture of soft-shelled turtles.

## Figures and Tables

**Table 1:** Nutritional composition of the basal commercial diets (% dry matter).

Crude fat	Crude protein	Crude ash	Crude fiber	Total phosphorus	Lysine
3.33	51.11	20.00	2.78	3.33	2.33

**Table 2:** Growth indexes of *P. sinensis* after 42 days of feeding with a commercial feed as a control group and a diet supplemented with *E. faecium* at 3.3 × 10^8^ CFU/g as treatment group.

Growth indexes/group	Control	Treatment
W_0_ (g)	107.8 ± 5.523	102.3 ± 7.446
Wt (g)	110.1 ± 5.403	104.4 ± 7.177
WGR (%)	2.272 ± 1.140	2.449 ± 1.251
SGR (%/d)	0.052 ± 0.026	0.056 ± 0.028
SR (%)	100	100

*Note:* Results are presented as the mean ± SE (*n* = 12).

Abbreviations: SGR, specific growth rate; SR, survival rate; WGR, weight gain rate; W_0_, initial weight; Wt, final weight.

## Data Availability

Data for this research article are available from the authors upon reasonable request.

## Supporting Information

Supporting Information The supporting information includes Table S1 as well as Figures S1–S5.
