# Supplementary material for: Effects of Dietary Supplement of Probiotic Enterococcus faecium on Intestinal Microbiota and Barrier Structure, Immune Function, and Antioxidant Capacity of Soft-Shelled Turtle Pelodiscus sinensis
Source: Aquac Nutr. 2025 Feb 7;2025:8066906. doi: 10.1155/anu/8066906 (PMC11828652; doi:10.1155/anu/8066906)
Supplement: Supporting Information — The supporting information includes Table S1 as well as Figures S1–S5. [file 8066906.f1.docx]

**Table S1** Sequence information of twenty samples in the experiment

| **Sample** | **Raw Reads** | **Clean Reads** | **Base(nt)** | **Avglen(nt)** | **GC** | **Q20** | **Q30** |
| --- | --- | --- | --- | --- | --- | --- | --- |
| Con1 | 105060 | 100677 | 42173312 | 418.9 | 53.78% | 99.20% | 96.99% |
| Con2 | 106332 | 104337 | 42194647 | 404.41 | 48.73% | 99.31% | 97.33% |
| Con3 | 104222 | 98148 | 41060597 | 418.35 | 51.99% | 99.18% | 96.97% |
| Con4 | 106150 | 99498 | 41605662 | 418.16 | 49.59% | 99.17% | 96.90% |
| Con5 | 105157 | 99506 | 41325152 | 415.3 | 49.56% | 99.15% | 96.80% |
| Con6 | 66131 | 61383 | 25842154 | 421 | 53.35% | 99.22% | 96.96% |
| Con7 | 119207 | 115565 | 47431719 | 410.43 | 49.48% | 99.21% | 96.99% |
| Con8 | 104177 | 101652 | 41527303 | 408.52 | 49.28% | 99.34% | 97.46% |
| Con9 | 107439 | 102276 | 43094432 | 421.35 | 51.57% | 99.16% | 96.81% |
| Con10 | 105781 | 102772 | 42949252 | 417.91 | 50.97% | 99.24% | 97.07% |
| Treat1 | 106347 | 101826 | 42079787 | 413.25 | 49.27% | 99.19% | 96.93% |
| Treat2 | 119870 | 117217 | 50233026 | 428.55 | 45.03% | 99.16% | 96.66% |
| Treat3 | 105975 | 97261 | 39743631 | 408.63 | 50.08% | 99.25% | 97.14% |
| Treat4 | 118690 | 111503 | 45854909 | 411.24 | 50.73% | 99.30% | 97.25% |
| Treat5 | 104133 | 92857 | 37853177 | 407.65 | 52.61% | 99.27% | 97.23% |
| Treat6 | 117505 | 110519 | 45276813 | 409.67 | 50.65% | 99.13% | 96.65% |
| Treat7 | 105313 | 98917 | 40503236 | 409.47 | 52.94% | 99.27% | 97.21% |
| Treat8 | 103677 | 101328 | 41370446 | 408.28 | 51.16% | 99.21% | 97.02% |
| Treat9 | 104106 | 100464 | 41266046 | 410.75 | 50.20% | 99.31% | 97.32% |
| Treat10 | 62533 | 59056 | 25053148 | 424.23 | 53.62% | 99.26% | 97.14% |

Con1 to Con10 were 10 individuals of the control group; Treat 1 to Treat 10 were 10 individuals of the treatment group


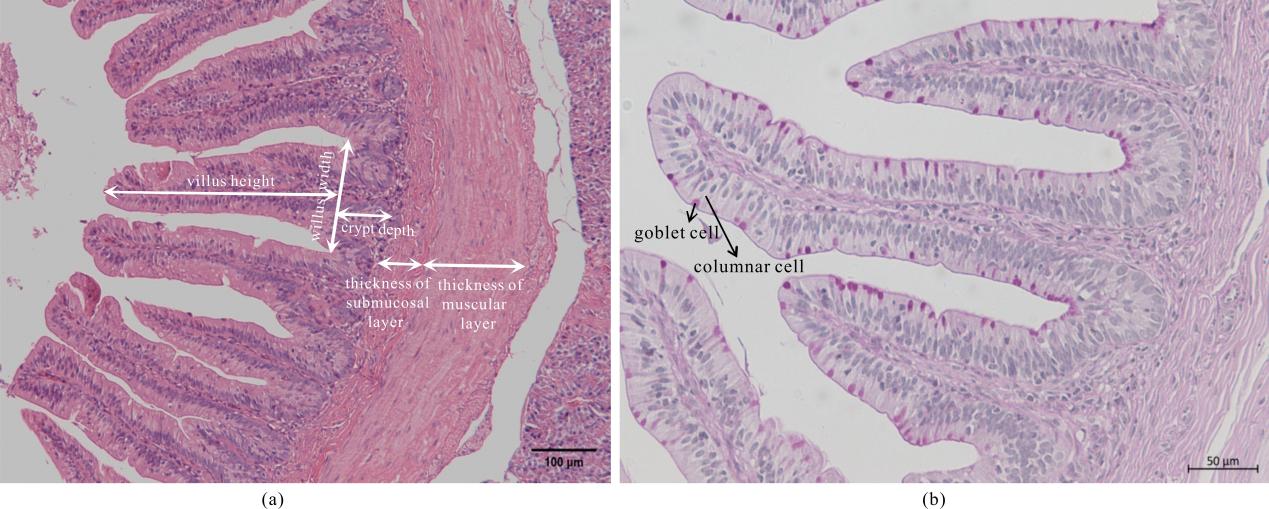


**Figure S1** Morphological indicators of the small intestine. (a)Villus height, villus width, crypt depth, thickness of submucosal and muscular layer are labeled in the section stained with HE (b)Goblet cell and columnar cell are labeled in the section stained with AB-PBS.


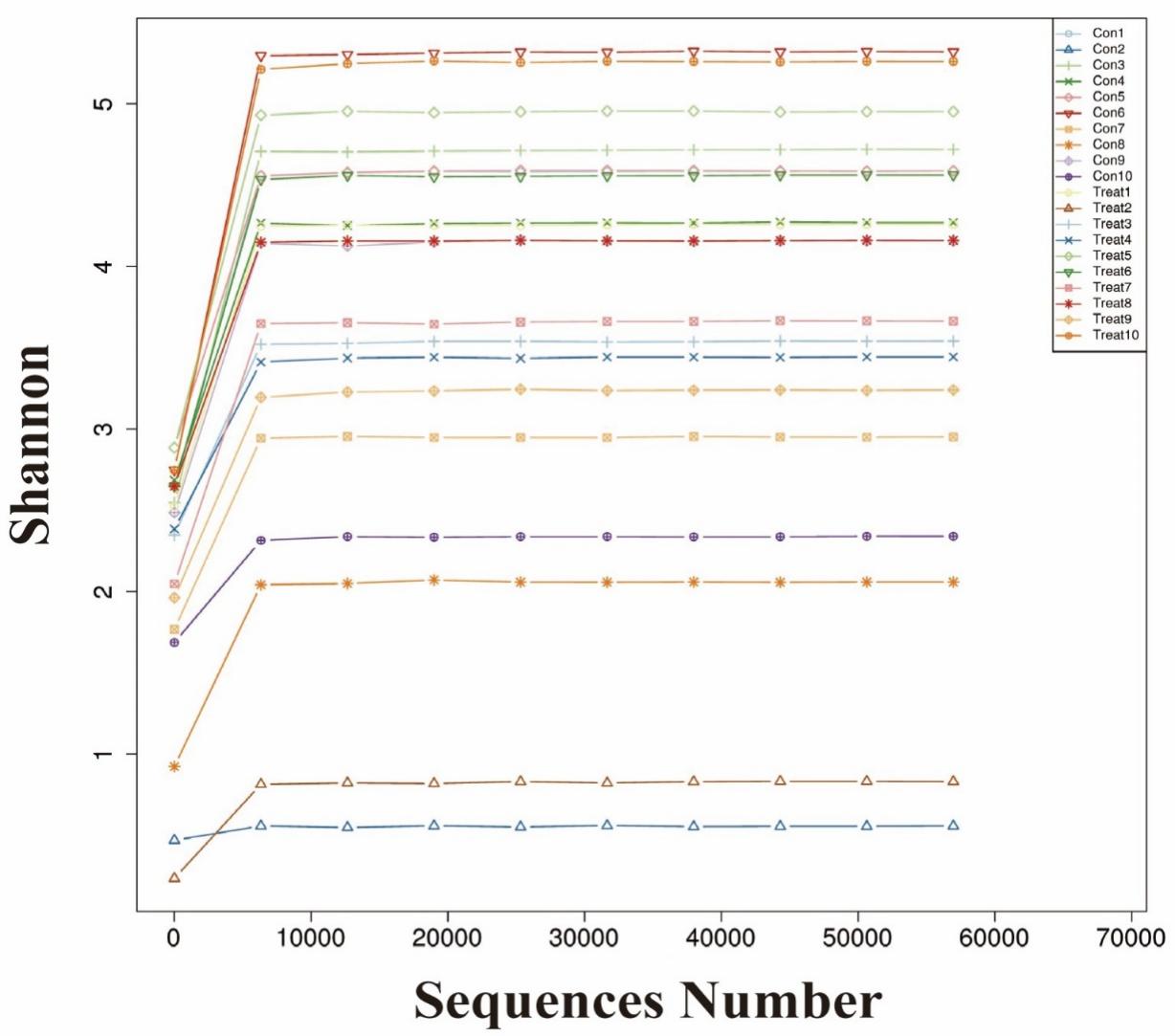


**Figure S2** Rarefaction curves of observed species number for the intestinal microbiome samples

**
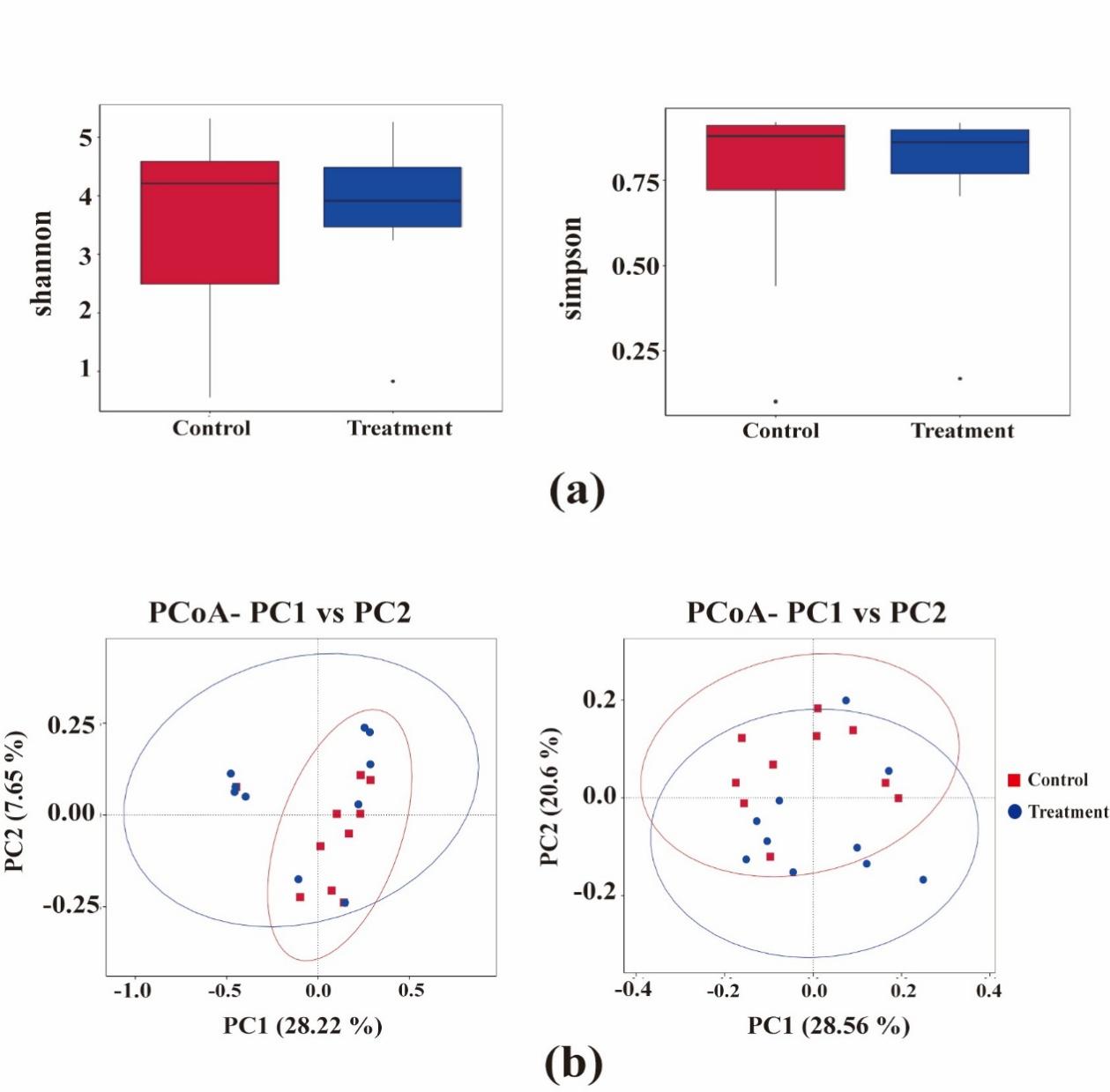
**

**Figure S3** Comparison of intestinal microbiota of the soft-shelled turtles treated with different diets. (a) *α*-diversity indexes (Shannon and Simpson) of the intestinal microbiota. Horizontal line represents the median of the data set, which is the middle value when the data are ordered from smallest to largest. Vertical Lines extend from the edges of the box to the smallest and largest values within a specified range. (b) Principal Co-ordinates Analysis (PCoA) based on weighted (left) and unweighted UniFrac distances (right).


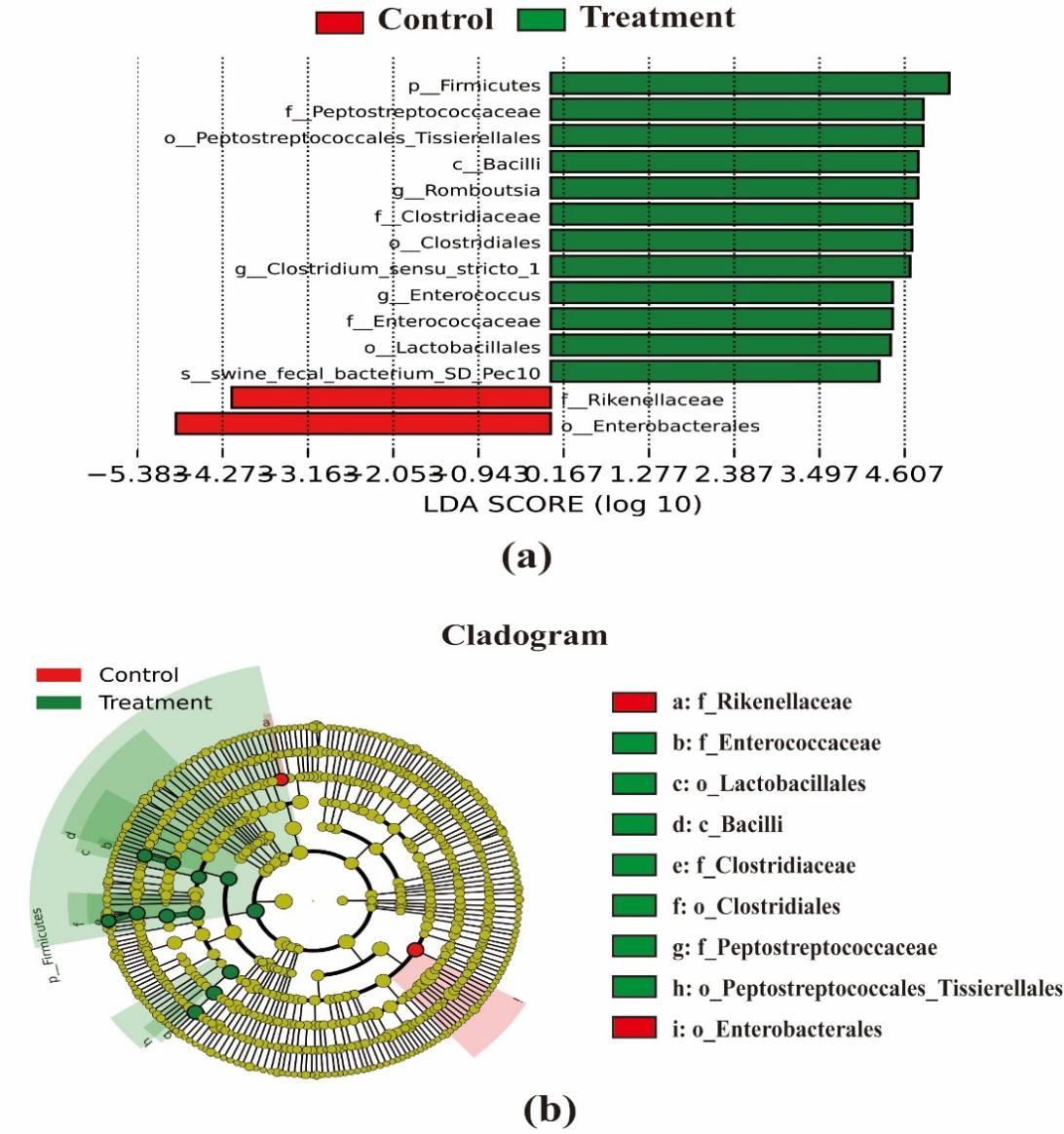


**Figure S4** The taxonomic cladogram and the LDA (linear discriminant analysis) score obtained from linear discriminant analysis effect size (LEfSe) analysis of the intestinal microbiota.


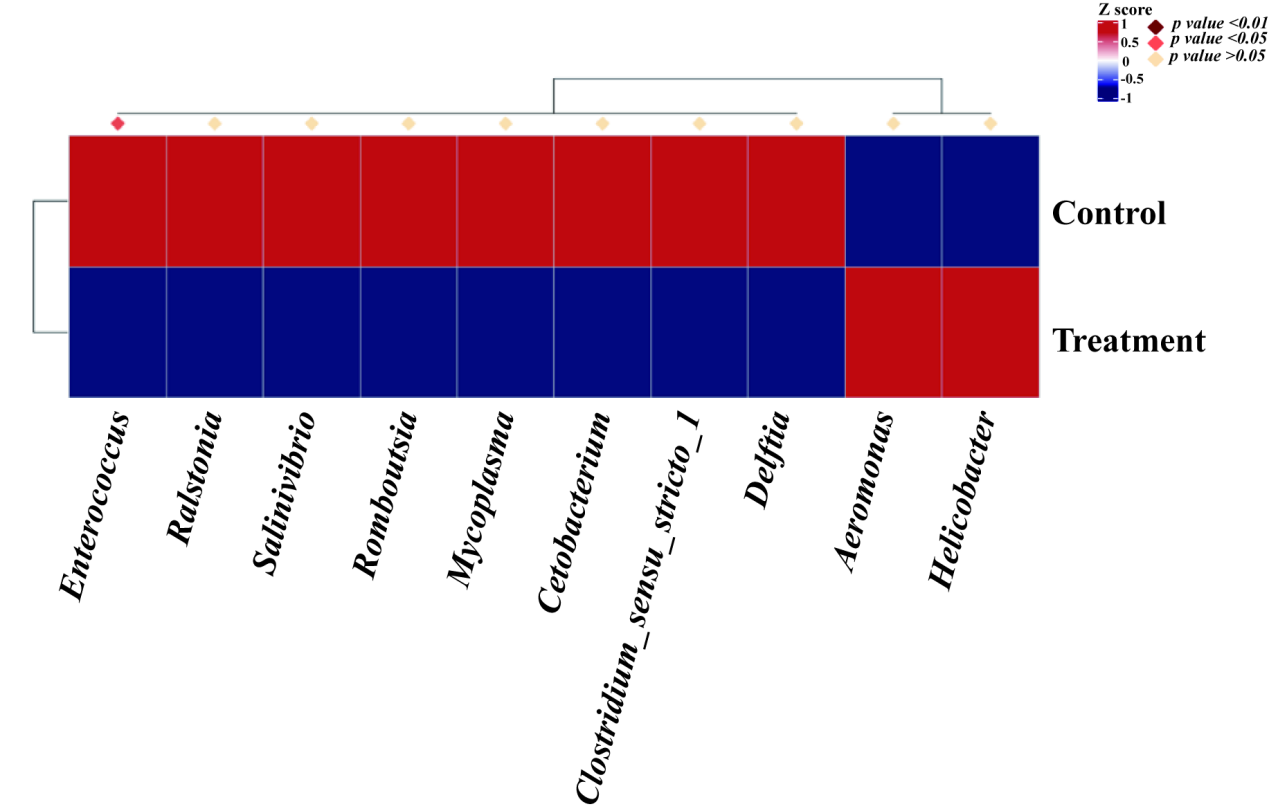


**Figure S5** Results of heatmap analysis for top 10 abundance species at genus level. The values represented in the heatmap are z-scores obtained by normalizing the relative abundances of the species.
